# Some Scientific Aspects of Insanity

**Published:** 1892-02-13

**Authors:** 


### Some Scientific Aspects of Insanity.

### XII.—GENERAL PARALYSIS.

General paralysis may be described as one of the most formidable nervous disease to which mankind is subject, because, so far as is known, no individual ever afflicted with it has recovered.

Although a minority of cases do not conform to this division of the disease into stages we may safely state that there are generally three stages of the disease most commonly observed: (1) a stage of mania simple in its nature, with tremulousness of the muscles of the mouth and expression; (2) a stage of delusional mania, with general unsteadiness in the movements of all the muscles of the body; (3) a stage of dementia, with paralysis of movement.

1. First stage.—The patient is usually excited, although he may be melancholic or confused and demented throughout the whole course of the disease. When the disease occurs in the female sex it is very seldom that maniacal symptoms show themselves, the patient being usually melancholic or demented. However, the most usual type met with in asylums in the first stage is mania with an appearance of great bodily vigour unbacked by any stamina or reserve power, and the expression of delusions of exaltation and power. The patient often says that he is strong enough to defeat ten men, to raise a ton weight with his teeth, to beat the record of the fastest runner, and many similar delusions. While he is speaking, those in the habit of observing such cases can always detect a slight trembling of the muscles of the lips and face, more especially when he attempts to pronounce long and difficult words, such as "conflagration," "constitution," "perambulator."

Patients in this stage are not as a rule troublesome, they are easily managed with a little tact on the part of their attendants. It often happens, that if refused a trifling request they burst into a passion of furious anger, but it is of the most transient nature, and the patient is very easily diverted into good humour again.

2. In the second stage the delusions of the patient become much more pronounced. He talks of being possessed of untold wealth, of shovelling gold with a spade. He talks of millions as if they were units, and he proposes feats of engineering which are inconceivable. The firm nutrition of the first stage passes away and the patient becomes flat and flabby. His gait becomes unsteady, and his speech so interrupted and laboured, that in pronouncing such words as those instanced above he only is able to slur them over with difficulty or to pronounce them in an altogether indistinguishable way. He is always in danger of falling on account of his unsteadiness, and the dangers attending a bad fall in the case of general paralytics are very grave owing to the fact that their bones are peculiarly brittle.

Without exception, all patients in the second stage of general paralysis tend to bolt their food, some of them in such a manner as to render choking almost inevitable. It therefore becomes the imperative duty of the attendant who has charge of the patient during meals to guard against such articles as hard crusts of bread, gristle, pieces of bone, or uncooked vegetables being mixed with the patient's food.

Greater care has to be observed in this stage with regard to the patients' habits, as they are apt to become very uncleanly. They also tend, especially at night, to tear their clothing, and it is no unfrequent thing to find in the morning that the general paralytic patient has, during the night hours, torn his blankets into thin strips, and wound them into ropes, while he has smeared his body with his own filth.

It is towards the end of this stage that convulsions usually make their appearance in these cases. The convulsions in general paralysis do not conform to the description already given of true epileptic fits. They are therefore called epileptiform convulsions. It frequently happens that an epileptiform convulsion may so closely resemble an epileptic fit as to be indistinguishable from it. As a general rule, however, epileptiform attacks are irregular, not only in different people, but often in the same subject. The stage of clonic convulsion is more prolonged, and there is generally unconsciousness, followed by coma.

The third stage of general paralysis is characterised by increased feebleness in co-ordination, and very often with the loss of the power of articulate speech. There is general emaciation and wasting of flesh, until ultimately the subject becomes little more than a skeleton enclosed in skin, the extremities are cold and lifeless, and there is a general and marked lowering of all the vital functions of the body. Notwithstanding these evidences of decay, the patient's appetite usually remains, and he continues to eat with much of the avidity for food which he evinced in the first and second stages. So lowered do the trophic functions become that bed-sores are apt to occur at all the bony promontories of the body where the skin is more tightly drawn over a projecting bone. Thus they frequently occur on the hips, on the buttocks, on the elbows, on the ankles, and if the patient is in bed they are most liable to occur on that part of the body most frequently and closely in contact with the mattress.

The care and treatment of general paralysis is too wide a subject to treat of within the scope of these lectures. It naturally divides itself into the care of the patient during the course of the three stages. In the first stage, where the patient labours under a more or less simple form of mania, the ordinary directions which have been indicated for the care of that disease will be sufficient. There are one or two points, however, on which it is well that a skilled attendant upon the insane should be informed. The first is a danger frequently observed in the very early part of the first stage, namely, a tendency to impulsive acts. These impulsive acts are often performed in the absence of excitement, and without any previous warning. They may take any form, from the destruction of property to homicidal attempts. Secondly, and closely allied to this impulsive tendency is the danger to which some cases are exposed of self-mutilation. Thirdly, there is the constant factor of explosiveness of temper upon the slightest and most trivial occasion. Attendants upon the insane should be able perfectly to recognise these outbursts of furious excitement. They are, with a little tact, extremely capable of being averted and changed into phases of good humour within a ridiculously short space of time. An attendant who loses his temper, or who comes into physical collision with one of these patients under the circumstances mentioned, is unfitted for his position, and should direct his attention to some other calling in life. In connection with the second stage there is first to be recognised the increased danger of choking at meals. Secondly, the ataxia and incoordination which renders it unsafe of the patient to perform work which he did in the first stage, such as carrying articles across slippery floors, or walking in dangerous places. Thirdly, there is the increased liability to fracture of bones, which may occur in this stage. It is, therefore, necessary that the patient should be handled with extreme caution; and that he should be protected from mingling over much with stronger patients. I will only add, by way of caution, that if possible no service requiring any degree of force should be performed for the patient by a less number than two or three attendants at a time. Fourthly, it has often been observed in this stage that there is a strong tendency on the part of the patient to steal and appropriate articles which are not his own. With regard to the treatment of the third stage, it is unnecessary here to dilate, as most of the points which would fall to be dealt with will be treated under the head of general nursing.

